# Texture and bio-functional characteristics of a Chinese steamed bread prepared from lotus root powder partially replacing wheat flour

**DOI:** 10.1038/s41598-021-95926-3

**Published:** 2021-08-11

**Authors:** Xiaoyue Li, Yuqiu Guo, Lirong Chen, Kaichang Liu, Kuijie Gong

**Affiliations:** 1 Crop Research Institute, Shandong Academy of Agricultural Sciences, Jinan, China; 2 Shandong Academy of Agricultural Sciences, Jinan, China

**Keywords:** Nutrition, Nutrition therapy

## Abstract

Making low GI of the Chinese steamed bread (CSB) with acceptable eating quality is a challenge. A CSB prepared from wheat flour partially substituted by lotus root powder (LRP) showed good prospects. RVA profile and texture profile were determined to evaluate the texture, while animal test were used to confirm the bio-functional attributes. The addition of LRP effectively changed the RVA profile of lotus-wheat incorporated flour (LWIF). CSB prepared from 30% LWIF showed acceptable eating quality with higher springiness, cohesiveness, and recovery while lower hardness. After 12 weeks of 30% LWIF administrating, the fast blood glucose of diabetic rat decreased from 17.6 to 5.8 mmol/L together with the reduction of serum TC, TG and LDL-C. The hepatic histopathological examination and serum levels changes of SOD, CAT and FFA confirmed LWIF could effectively protect the liver of the diabetic rats from damage caused by oxidative stress.

## Introduction

Lotus root (*Nelumbo nucifera*) is a very popular food native to some tropical Asian countries, and Australia^1,2^. The rhizome of lotus has been found to be rich in protein, starch, phosphorus, copper, potassium, manganese, vitamins C, B1, B2 and B6, while very low in saturated fat^1^. Furthermore, lotus root contains abundant dietary fiber, polyphenolic compounds, and polysaccharide^2–4^. Such health-beneficial properties as hypoglycemic, anti-allergy, anti-inflammatory, and antioxidant activities have been widely reported^2,3,5–8^.

Lotus root often used as vegetable for its hard and crispy texture, special aroma and mouth feel^9^. Lotus root powder (LRP) is another usual product which is consumed as breakfast, fast food, traditional confectionery and food additives by Asian people^1^. However, the consumption of LRP as the above form is limited for its infrequently eaten. Due to rich in starch and abundant functional components, health-benefit staple foods can be the important development way for LRP. However, there is less information about the application of LRP in staple foods.

Chinese steamed bread (CSB) is the principal foods for many people in the world^10^. Nowadays, steamed bread is steadily increasing for its comfortable texture. However, CSB has a high glycemic index (GI) which is about 86^10^, and not suitable for most hyperglycemia, insulin resistance, and metabolic syndrome population. Therefore, some functional ingredients such as sorghum flour, potato, sweet potato flour are used to substitute wheat flour to improve the health attributes^10–14^. However, there are no reports that low GI steamed bread can be obtained by adding cereal or rhizome flour.

Furthermore, functional ingredient addition tends to decrease the specific volume of CSB, while lead to weak taste such as increased hardness and chewiness^15^. Therefore, strategies to maintain the sensory quality while increasing the nutritional quality of CSB should be developed^10^.The selection of appropriate substitution level and methods to maintain or improve the textural and sensory quality has become an important research direction for the preparation of CSB with low GI. However, there are no related reports about LRP substitution together with texture evaluation of CSB can be obtained.

Therefore, the objectives of the present study were to evaluate the sensory and healthy beneficial value of CSB prepared from wheat flour partially substituted by LRP. Sensory related parameters such as pasting, textural properties of different substitution levels were investigated to select suitable proportion. In addition, glycemic index (GI) of CSB was calculated to conclude the substitution level further. Finally, animal experiments were conducted to assess the hypoglycemic, hypolipidemic and antioxidative stress effect of CSB prepared from wheat flour partially substituted by LRP. We want to provide a CSB with good sensory and bio-functional attributes to help the treatment of chronic disease.

## Materials and methods

### Material

Lotus root powder was contributed by Dongying Shengyuan agricultural ecosystem Co., Ltd. Wheat flour (Yihaijiali, Qingdao, China) was purchased from a local market in Jinan, Shandong, China. All plant material is in compliance with Plant Material Collection Guidelines of Shandong Academy of Agriculutral Sciences of China. Streptozotocin (STZ) was purchased from Sigma Chemical Co. (St. Louis, MO, USA). All other reagents used were of analytical grade.

### Pasting properties determination of lotus-wheat incorporated flour

Formulations of lotus-wheat incorporating flour (LWIF) were composed of 100% wheat flour (WF) as control, or by substituting lotus root powder (LRP) for WF at 10%, 20%, 30%, and 40% (w/w). Pasting property was determined according to Muna et al.^16^ with some modification. Suspensions were prepared by 3 g WF or LWIF in 25 mL distilled water and analyzed by a Rapid Visco Analyzer (RVA, model RVA3D, Newport Scientific Instrument & Engineering, NSW, Australia). The slurry was held at 50 °C for 1 min, heated to 95 °C in 3.75 min with a fixed speed of 60 rpm and then at 95 °C for 2.50 min. Then, it was cooled down to 50 °C for3.75 min and held at 50 °C for 2 min. The data were recorded as the average of duplicate measurements.

### Texture analysisof lotus-wheat steamed bread

The texture profile of steamed bread was analysed according to previous reports^14,16^ using a TA-XT Plus texture analyser (Stable Micro Systems, Goldalming, UK) equipped with a P/36R cylindrical probe. Two slices of 25 mm thickness were cut from each loaf of steamed bread for testing. Instrument were set in TPA mode and the test parameters as follows: pretest speed of 2.0 mm/s, test speed of 1.0 mm/s, posttest speed of 1.0 mm/s, deformation level of up to 50%, deformation time interval of 5.0 s, trigger type of AUTO, starting point induction force of 5 g, data acquisition rate of 200pps. From the TPA experimental curve, six parameter values could be obtained: hardness, elasticity, cohesiveness, adhesiveness, chewiness, and recovery.

### Animal test

Wistar male rats weighing 250 ± 20 g were obtained from the Experimental Animal Centre of Shandong University (Jinan, China). All animals were kept in an environmentally controlled room with a natural light–dark cycle. The animals had free access to water and were fed with standard laboratory diet. The study was approved by Preventive Medicine Ethics Committee of Shandong University and was carried out in compliance with the ARRIVE (Animal Research: Reporting of In Vivo Experiments) guidelines.

### Modeling and feeding of experimental animals

Rats were adaptively fed for one week before modeling and randomly divided into 3 groups (n = 8). 1 normal control group fed with basic diet. The other 2 model groups were fed with high fat and sugar diet. The composition of the experimental diet was shown in Table 1. The test group diet main composition was 30% LWIF according to the result of texture analysis. All diets were prepared by Beijing Huafukang Biological Technology Co., Ltd. The model rats were induced with high fat and sugar diet and STZ injection. STZ can destroy islet β cells of rats, increase the oxidative invasion and induce diabetes in animals. After 4 weeks of administration, fast for 8–10 h. The model rats were injected intraperitoneally with STZ at 45 mg/kg (STZ dissolved in 0.1 citrate buffer with the STZ concentration was of 1%). After fasting for 72 h, fast for 8–10 h and then measured blood glucose. Rats with fasting blood glucose (FBG) ≥ 11.1 mmol/L were used as the modeling standard. Modeling rats were divided into two groups according to FBG. Model control group continued to feed high fat diets, while test group was fed with tested diet. The animals had free access to water and diet. FBG was measured every 2 weeks at 4–8 week and once a week at 8–12 week.

**Table 1:** Composition of experimental diets (g/100 g diet).

Ingredients	Normal	High Fat Diet	Tested	Ingredients	Normal Diet	High Fat	Tested
Corn flour	41	27.5	41	LWIF	–	–	35
Wheat bran powder	15	10	–	Sucrose	–	20	–
Soybean meal	20	13	20	Lard	–	10	–
Fish meal	2	1.5	2	Cholesterol	–	2.5	–
Bone meal	2	1.5	2	Sodium cholate	–	0.5	–
Wheat flour	20	13	–	Seasame oil	–	0.5	–

### Sample collection and determination of experimental animals

Rats were fasted for 12 h and then were anesthetized with 3% sodium pentobarbital before sacrifice at the end of 12 weeks. Whole blood of 2 mL was taken from the abdominal aorta and added to test tubes in which anticoagulants were placed in advance. Chromatographic determination of HBA1c was carried out. The remaining blood was centrifuged at 3000 r/min for 15 min. The serum TC, TG, HDL-C, LDL-C, T-SOD, CAT, FFA, and hs-CRP contents were determined according to the instructions of the analysis kit. Total cholesterol assay kit, triglyceride assay kit, high-density lipoprotein cholesterol assay kit, low-density lipoprotein cholesterol assay kit, total superoxide dismutaseassay kit (hydroxylamine method), catalase assay kit (visible light) and high-sensitivity C -reactive protein assay Kit was purchased from Nanjing Jiancheng Bioengineering Institute (Nanjing, China).

The hepatic of each group of rats were quickly taken after blood collection and rinsed with physiological saline. A part of the hepatic homogenate was prepared to measure the MDA content. The remainder was fixed with tissue fixation solution, and liver HE staining was performed. The histopathological characters were observed and recorded under HM 325 colorized pathology image analyzer (Thermo Scientific, Waltham, MA, USA).

All of the experimental procedures were performed in accordance with the guidelines issued by the Shandong University and conformed to the Guide for the Care and Use of Laboratory Animals published by the US National Institutes of Health.

### Statistical analysis

All the statistical analyses comprised one-way analysis of variance (ANOVA) using SAS 9.2 statistical software (SAS Institute Inc., USA). The data presented were the means of three experiments, along with the standard error of the mean. The means were compared by Fisher's least significant difference (LSD) test, and differences at *P* < 0.05 were considered significant.

## Results and discussion

### Pasting properties of wheat flour partially substituted with lotus root powder

**Figure 1:**
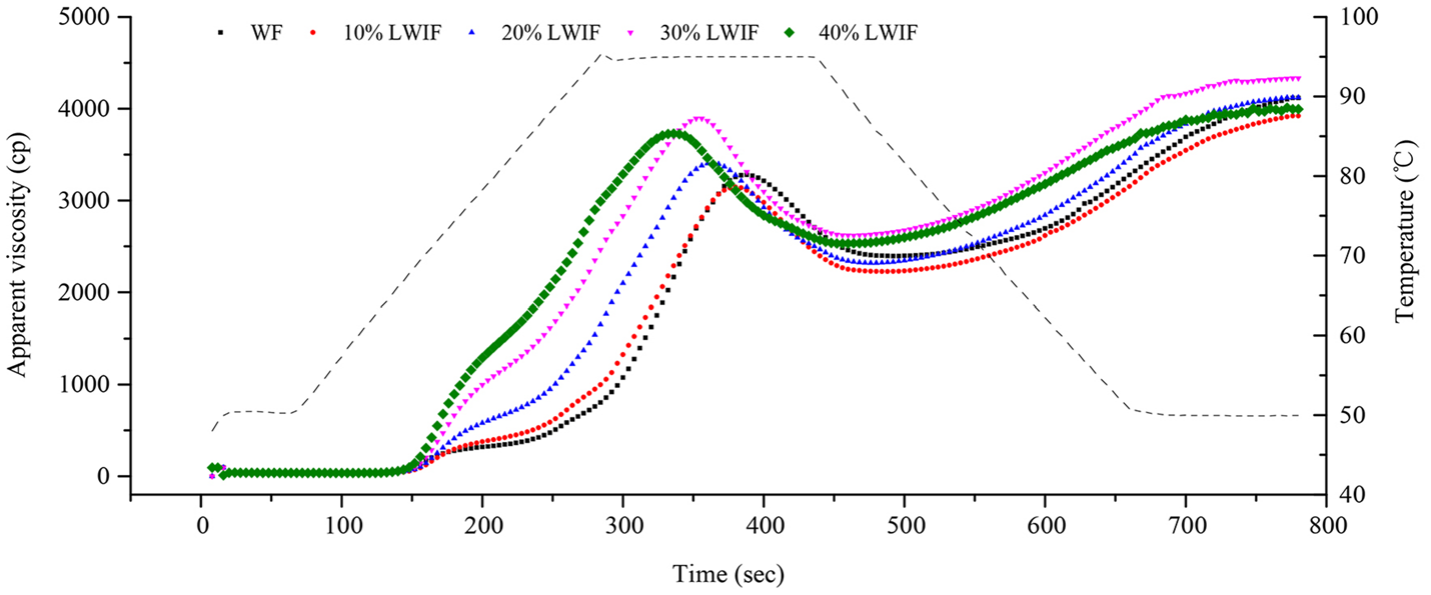
RVA profile of WF and LWIF of different substitution level.

The pasting characteristics of starch are indispensable for assessing the quality of foodstuffs, notably in modifying texture and improving stability of starch-based food products^17^. The addition of LRP effectively changed the RVA profile of LWIF compared with WF (Fig. 1). The peak viscosity (PV), through viscosity (TV) and final viscosity (FV) all decreased at 10%, reached the highest at 30% and decreased again at substitution level of 40%. Some differences with the above were that the setback viscosity (SV) reached the highest at 20% level. The higher PV during heating indicated the higher ability of the starch granules to swell before rupture^18^. Greater PV implied thickening effects in starch pastes^19^. Final viscosity was associated with the starch tendency to retrograde during cooling^20^. In summary, the highest PV and FV of 30% LWIF meant high degree of swelling and ability to form strong gels. The higher setback viscosity of starches was indicative of lower stability of cool starch paste or the faster retrogradation of starch paste^21^. The 30% LWIF had the equivalent SV with WF meant that it possessed better anti-retrogradation ability and could be served as a symbol of good cooking quality^22^. Therefore, the substitution level of 30% was more potent for preparing good eating quality foods.

### Texture properties of steamed bread

**Figure 2:**
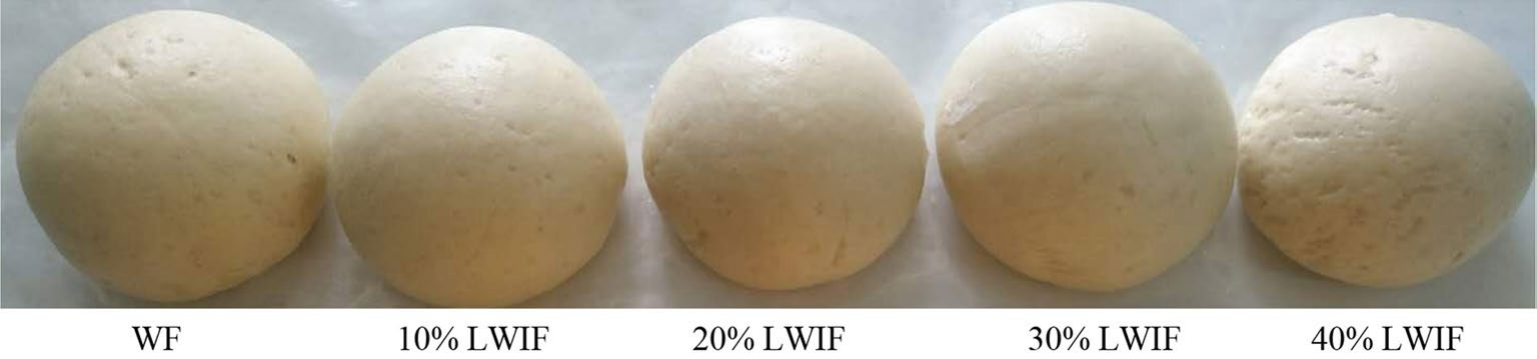
Steamed bread prepared from WF and LWIF of different substitution level.

The CSB prepared from wheat flour substituted with different level of LRP was presented in Fig. 2. The texture characteristic is an indicator to assess the sense of chewing food in the mouth^16,19,23^. The textural characteristics of fresh steamed bread were presented in Table 2. Hardness was significantly (P < 0.05) increased due to the substitution of LRP. However, 30% LWIF showed similar hardness to WF, whereas no significant (P > 0.05) differences were observed among other formulations. Hardness is generally used as a major indicator of textural properties. 30%LWIF showed similar hardness to WF. The greater the hardness, the more firmer is the crumb^19,24^. The chewiness showed similar trend to hardness as previous report^25,26^, indicating their positive correlation between hardness and chewiness. Obviously, 30% LWIF exhibited more fluffy texture, which was closer to WF. The springiness, cohesiveness, and recovery all increased with the substitution level increase (Table 2). Springiness is associated with the number of air bubbles and cohesiveness is related to the density and energy required to chew the food^27^. Cohesiveness is indicative of the strength of internal bonds making up the bread crumb^28^. Cao et al.^13^ concluded that lower hardness and chewiness, higher springiness and cohesiveness improved the quality of steamed bread byincreasing the stability of gluten network structure and gas-holding capacity of the dough. Luo et al.^29^ indicated that lower recovery means that the steamed bread is easier to be damaged and deformed. In summary, higher springiness, cohesiveness, and recovery meant higherquality of the steamed bread from the previous reports. In present study, 40% LWIF exhibited the highest value of the three. Considering that 40%LWIF possessed higher hardness and chewiness, the substitution level of 30% might be an appropriate choice.

**Table 2:** Texture of fresh steamed bread prepared from WF and LWIF.

Formulation	Hardness (g)	Springiness (min)	cohesiveness (min)	chewiness (BU)	recovery (v.v.)
WF	1727.545 ± 73.336b	0.848 ± 0.008d	0.816 ± 0.001 d	1265.510 ± 47.369b	0.470 ± 0.014c
10%LWIF	2103.705 ± 202.803a	0.862 ± 0.022 cd	0.824 ± 0.029 cd	1404.706 ± 177.770b	0.457 ± 0.025c
20%LWIF	2180.258 ± 98.269a	0.889 ± 0.012bc	0.836 ± 0.005 c	1601.433 ± 47.210a	12 ± 0.003b
30%LWIF	1809.047 ± 69.533b	0.916 ± 0.009ab	0.864 ± 0.004 b	1272.696 ± 60.198b	0.551 ± 0.008a
40%LWIF	2151.726 ± 269.760a	0.954 ± 0.033a	0.88 ± 0.003 a	1799.173 ± 280.595 a	0.572 ± 0.008a

Data are mean values ± standard errors of three replicates. Mean values followed by the different letter in the same row are significantly difference (*P* < 0.05).

### Effect of LWIF administration on blood glucose response of diabetic rats

**Figure 3:**
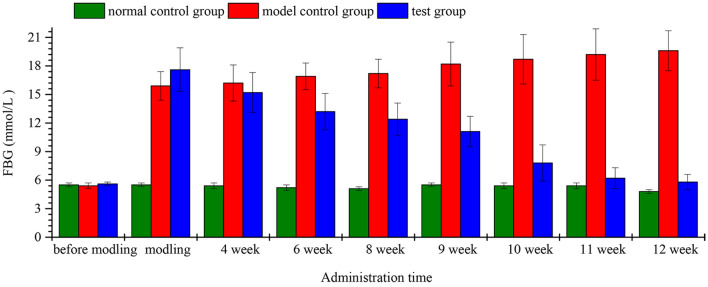
FBG changes of rats with different diets. Data are mean values ± standard errors of three replicates.

The FBG changes of the rats in each group were presented in Fig. 3, which was ranged from 5.1–5.7 mmol/ L before the modeling. The FBG of the model control group and the test group were higher than 11.1 mmol/L after the modeling, indicating that the modeling was successful. After 4 weeks of feeding, the FBG in the test group dropped from 17.6 to 15.2 mmol/L. During 4–12 week, the FBG in changes of rats with different diets. Data are mean values ± standard errors of three replicates. The model control group continued to increase, while it continued to decrease in the test group. After 12 weeks administration, the FBG in the test group decreased to about 5.8 mmol/L, suggesting that long-term feeding of LWIF could reduce FBG in diabetic rats. The reason may be due to functional materials in LRP such as polyphenol^3^ and polysaccharides^30,31^. Park et al.^6^ concluded that the hypoglycemic effects of lotus root performed via an insulin-like action, insulin sensitizing ability, and α-glucosidase inhibitory activity. In addition, another factor was that the granular, gelatinized and retrograded lotus root starches were middle-GI foods^7^.

Furthermore, changes of serum levels of glycosylaoted hemoglobulin A1c (HbA1c) also confirmed the response in test group. After 12 weeks of administration, the HbA1c level (8.98 ± 1.58%) of the test group rats was significantly (P < 0.05) lower than the model control group (11.23 ± 1.63%) and equally with normal control group (8.65 ± 1.39%). Measurement of the HbA1c value is commonly used to assess long-term diabetic control in laboratory^32^. These results suggested that LWIF had hypoglycemic effects.

### Effect of LWIF administration on serum lipid level

Compared with the normal control group, the serum TC, TG and LDL-C contents of the model control group were significantly increased (*P* < 0.05), and the HDL-C contents were significantly (*P* < 0.05) reduced (Table 3). This demonstrates the dangers of a continuous high-fat and high-sucrose. It is clear that feeding LWIF to rats can reverse this hazard. The content of serum TC, TG and LDL-C in the test group was significantly lower than that in the model control group (*P* < 0.05), while the content of HDL-C increased significantly (*P* < 0.05). From Table 3, the diabetic rats fed with LWIF for 12 weeks, most serum lipid levels were close to the level of normal control group. Furthermore, the TG level in test group was lower than that in normal control group, while the HDL-C level was higher than the latter. Zhou et al.^32^ reported that the reduction of serum TG and increase of HDL-C was due to flavonoids extracted from lotus. These effects of flavonoids supplementation from lotus may be due to low activity of cholesterol biosynthesis enzymes and or low level of lipolysis which is under the control of insulin^33^. The results suggested that long-term LWIF administration improved the lipid metabolism of diabetic rats.

**Table 3:** Serum lipid level of rat fed with LWIF and control diet.

Group	TG (mmol/L)	TC (mmol/L)	HDL (mmol/L)	LDL (mmol/L)
Normal control	1.228 ± 0.342b	1.712 ± 0.443c	0.573 ± 0.177b	0.427 ± 0.158b
Model control	3.303 ± 0.365a	17.847 ± 1.498a	0.01 ± 0.015a	17.083 ± 1.158a
Test	0.763 ± 0.196c	2.253 ± 0.298b	0.915 ± 0.093c	0.510 ± 0.122b

Data are mean values ± standard errors of three replicates. Mean values followed by the different letter in the same row are significantly difference (*P* < 0.05).

### Effect of LWIF administration on hepatic and serum oxidative stress

Oxidative stress and free radical formation may lead to a range of pathological conditions such as diabetes, cancer, cardiovascular disease, etc.^11,34^ High fat diets would increase oxidative stress^35^, while dietary natural antioxidants strengthen the endogenous antioxidant system, thus reducing the oxidative stress^8^. The levels of SOD and CAT in the model control group were significantly (*P* < 0.05) lower than those in the normal control group and test group (Table 4). The latter two showed no significant difference between them, indicating that LWIF administration had significant improvement on antioxidative stress. The model control group also exhibited slightly higher hepatic MDA content. However, there were no significant differences among them.

**Table 4:** Serum oxidative stress of rat fed with LWIF and control diet.

Group	SOD (U/mL)	MDA(nmol/g)	CAT(nmol/min/mL)	hs-CRP (μg/L)	FFA (umol/L)
Normal control	3.713 ± 0.378a	9.929 ± 0.851a	11.383 ± 2.584a	55.224 ± 14.583a	1084.167 ± 74.178b
Model control	1.573 ± 0.394b	11.623 ± 1.808a	6.655 ± 0.325b	60.779 ± 10.238a	1229.667 ± 54.193a
Test	2.385 ± 0.899a	10.443 ± 1.103a	10.328 ± 1.280a	50.136 ± 8.797a	721.000 ± 105.272c

Data are mean values ± standard errors of three replicates. Mean values followed by the different letter in the same row are significantly difference (*P* < 0.05).

High sensitivity C-reactive protein (hs-CRP) is a systemic, non-specific inflammatory marker associated with insulin resistance and metabolic syndrome^36^. In present study, tested diet showed slightly lower hs-CRP level, but no significant (*P* > 0.05) differences in hs-CRP of the three (Table 4). Elevated free fatty acids (FFAs) resulted in increased oxidative stress in a variety of tissues and contribute to insulin resistance^37^. Administration of LWIF for 12 weeks was helpful for suppressing oxidative stress by decreasing the levels of FFA (Table 4). Furthermore, serum FFA content of rats in test group was only 721.000 ± 105.272 μmol/L, evenly lower (*P* < 0.05) than that in normal control group. This indicated that LWIF could improve the body's antioxidant defense system and protect the body from oxidative stress.

**Figure 4:**
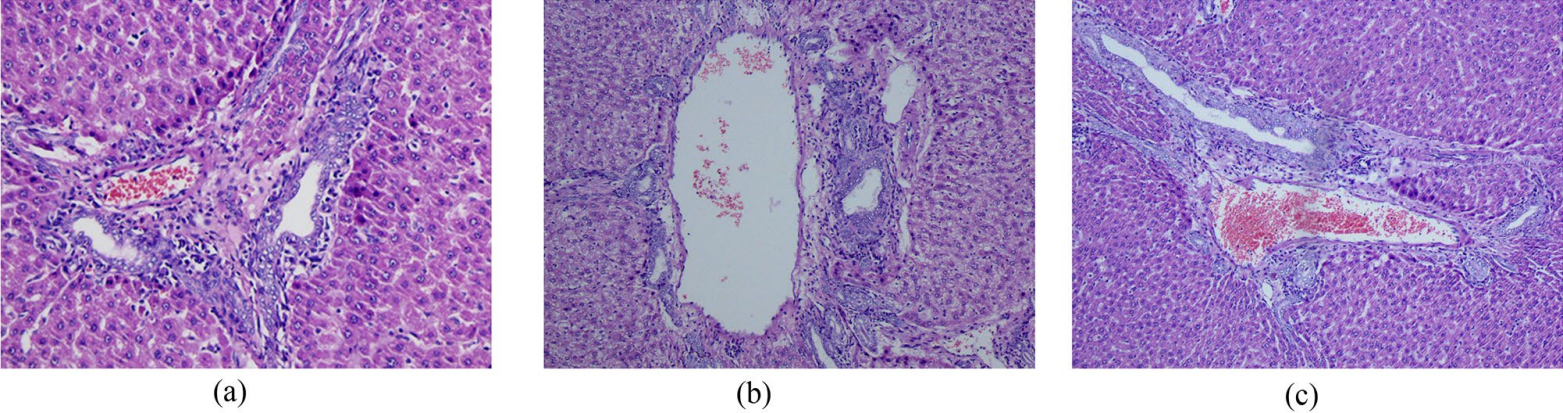
Hepatic histopathological of rats of normal group (**a**), model control group (**b**) and test group (**c**).

The hepatic histopathological examination further confirmed the protective effect of the LWIF administration in the diabetic rats (Fig. 4). There were no pathological changes in the livers in the normal control group (Fig. 4a). However, the model control group showed a small number of hepatocytes steatosis like balloon changes compared with the normal control group and the test group. Arteriolar mild vitreous degeneration could be observed from the model control group (Fig. 4b). After 12 weeks of administration, the apoptosis of the diabetic rats was significantly reduced, and the infiltration of inflammatory cells was significantly reduced (Fig. 4c). This illustrated that LWIF could effectively protect the liver from damage caused by oxidative stress.

## Conclusions

Lotus root powder incorpated with wheat flour could prepare Chinese steamed bread with good sensory and bio-functional attributes. CSB prepared from 30% LWIF showed higher springiness, cohesiveness, and recovery and fluffy texture, which possessed good eating quality. After 12 weeks of 30% LWIF administration, average FBG of diabetic rats dropped from 17.6 to 5.8 mmol/L, while serum lipid of TG, TC, LDL, and HDL were close to the level of normal control group. Furthermore, it could improve serum SOD and CAT level, reduce serum FFA content, and reduce hepatocyteapoptosis and infiltration of inflammatory cells. Therefore, 30% LWIF possessed good hypoglycemic, hypolipidemic and antioxidative stress effect. CSB prepared from wheat flour substituted by 30% LRP will be a promising food product especially in complementary treatment of chronic disease.
